# Supplementary material for: Mutant alleles differentially shape fitness and other complex traits in cattle
Source: Commun Biol. 2021 Dec 2;4:1353. doi: 10.1038/s42003-021-02874-9 (PMC8640064; doi:10.1038/s42003-021-02874-9)
Supplement: Supplementary file 2 — Supplementary Information [file 42003_2021_2874_MOESM2_ESM.pdf]

Supplementary information of:

Mutant alleles differentially shape fitness and other complex traits in cattle

Ruidong Xiang[1,2], Ed J. Breen[2], Sunduimijid Bolormaa[2], Christy J. Vander Jagt[2], Amanda J. Chamberlain[2], Iona M. Macleod[2], Michael E. Goddard[1,2]

[1] *Faculty of Veterinary & Agricultural Science, The University of Melbourne, Parkville 3052, Victoria, Australia*

[2] *Agriculture Victoria, AgriBio, Centre for AgriBiosciences, Bundoora, Victoria 3083, Australia.*

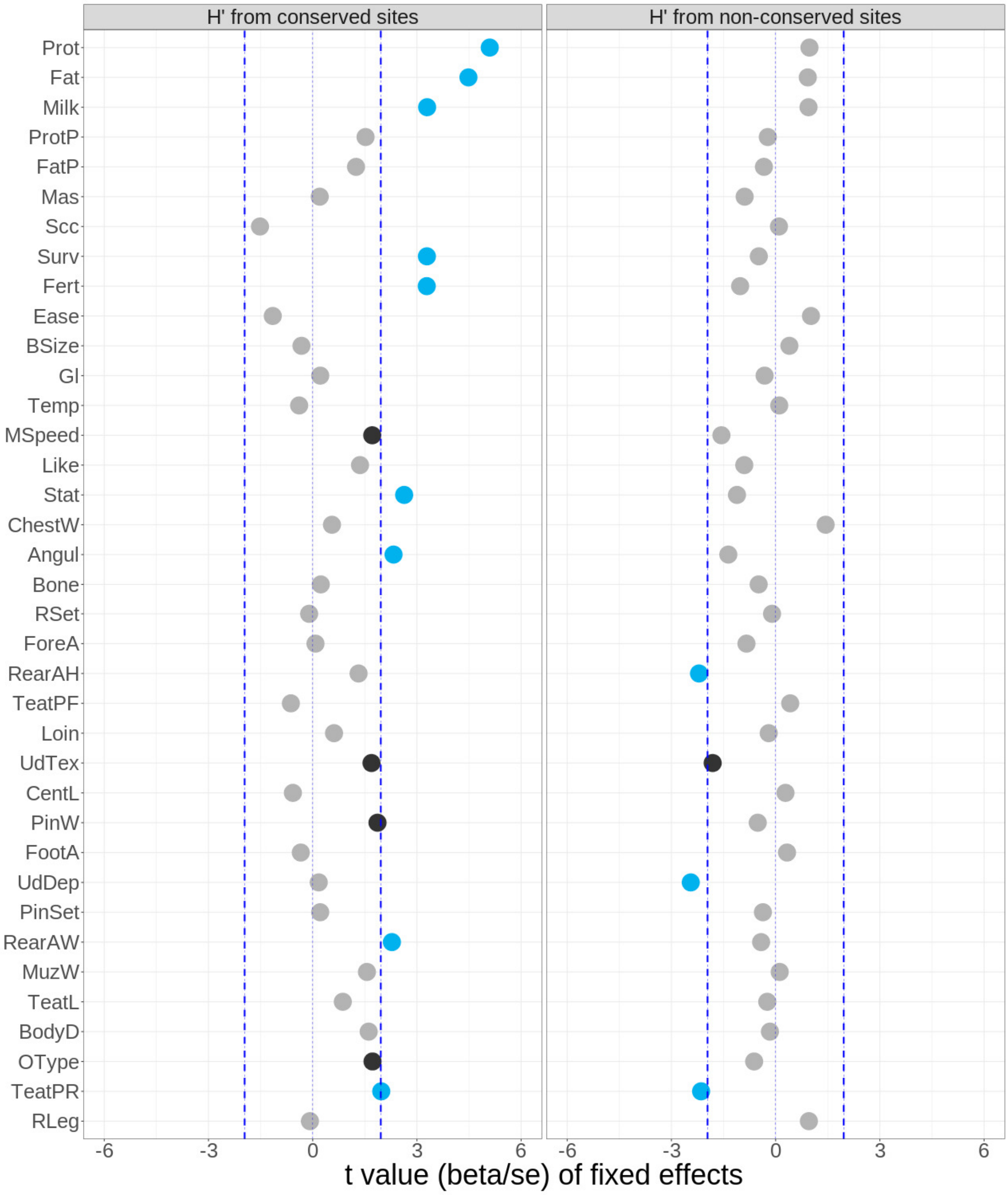

Supplementary Figure 1. Fixed effects of genomic heterosis (H') of individuals on 37 cow traits (N>100k). The beta values and standard errors for each trait were generated using GREML, fitting a covariate representing H' from 317,279 conserved sites and fitting another covariate representing H' from the remaining 15,718,164 sites together with other fixed effects. Blue dashed lines indicate t value of -1.96 and 1.96 commonly used to indicate the significance. Significant effects of conserved H' indicate that the trait is related to fitness. Blue dots indicate the H' with $p < 0.05$ while black dots indicate the H' with $0.05 \leqslant p < 0.1$.

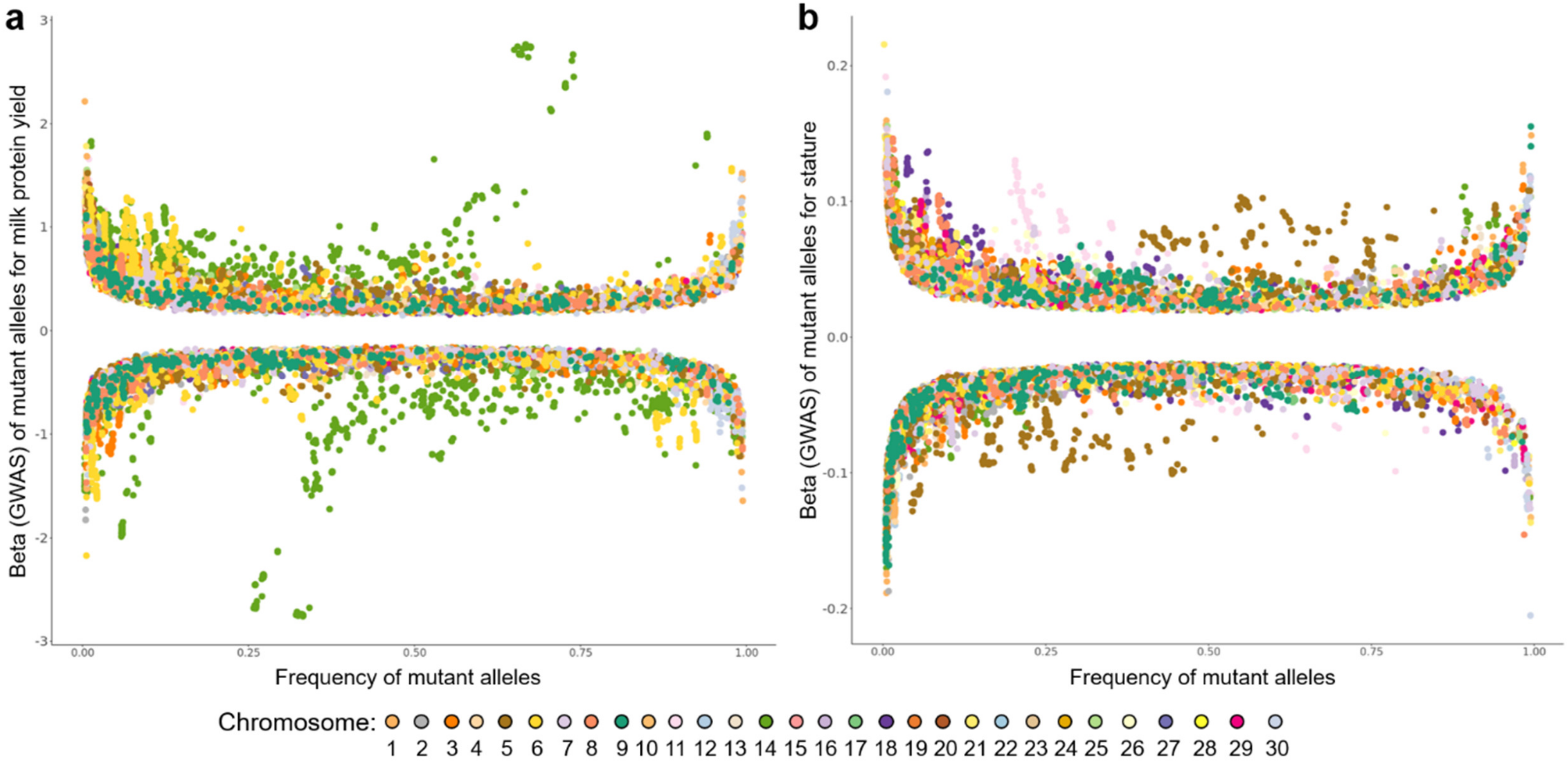

Supplementary Figure 2. Examples of the GWAS effects (beta) of mutant alleles versus their frequency for milk protein yield (**a**) and stature (**b**). Note that green dots (chromosome 14) in **a** were from the *DGAT1* locus, a known locus for cattle milk production traits and brown dots (chromosome 5) in **b** were those variants from *CCDN2* locus, a known locus for cattle stature. Variants with a p-value < 0.05 in both sexes are shown.

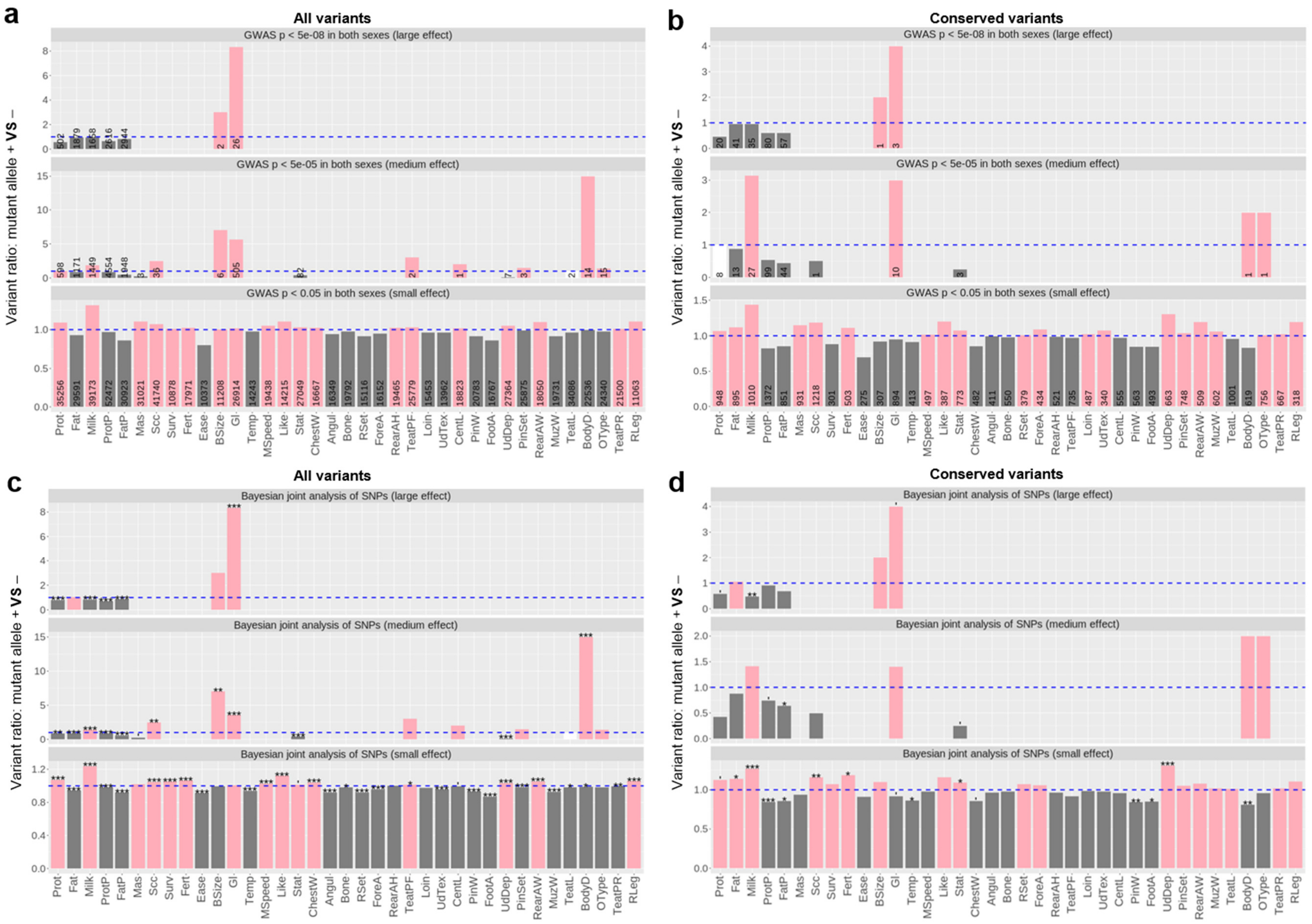
a
All variants
b
Conserved variants
c
All variants
d
Conserved variants
GWAS p < 5e-08 in both sexes (large effect)
GWAS p < 5e-05 in both sexes (medium effect)
GWAS p < 0.05 in both sexes (small effect)
Bayesian joint analysis of SNPs (large effect)
Bayesian joint analysis of SNPs (medium effect)
Bayesian joint analysis of SNPs (small effect)
Variant ratio: mutant allele + VS –
Prot Fat Milk ProtP FatP Mas Scc Surv Fert Ease BSize GI Temp MSpeed Like Stat ChestW Angul Bone RSet ForeA RearAH TeatPF Loin UdTex CentL PinW FootA UdDep PinSet RearAW MuzW TeatL BodyD OType TeatPR RLeg

Supplementary Figure 3. The ratio (y-axis) between the number of variants with mutant alleles (MAs) increasing the trait (+) and the number of variants with mutant alleles decreasing the trait (–). GWAS effects of MAs are shown for all variants (a) and variants from conserved sites (b). BayesR joint effects of MAs are shown for all variants (c) and variants from conserved sites (d). Pink colour: the majority of variants with MAs tend to increase the trait. Dark grey: the majority of variants with MAs tend to decrease the trait. Numbers in bars: total number of variants significant at the given threshold. Stars: p-value for the significance of the difference in the distribution of BayesR effects between ancestral and mutant alleles (c, d), '.': $p < 0.1$; '*': $p < 0.05$, '**': $p < 0.01$, '***' $p < 0.001$.

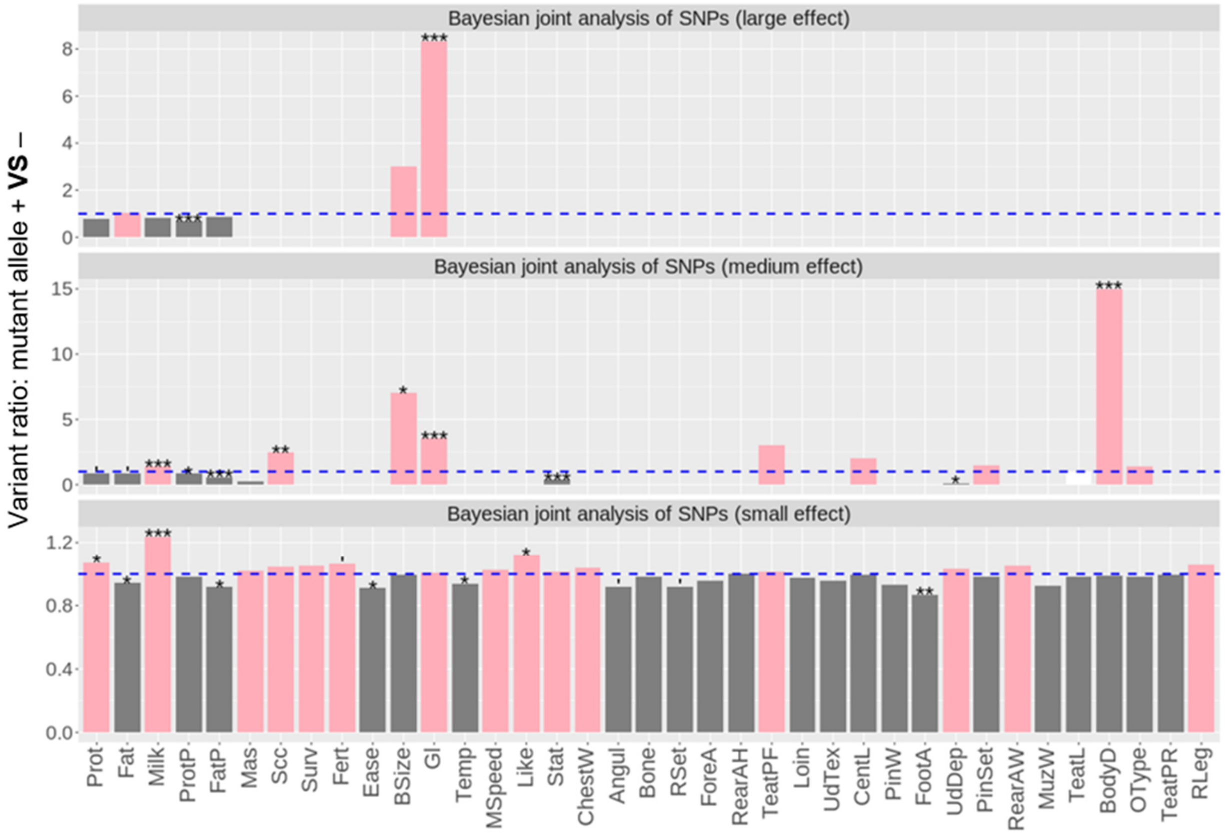

Supplementary Figure 4. The ratio (y-axis) between the number of variants with mutant alleles (MAs) increasing the trait (+) and the number of variants with mutant alleles decreasing the selected trait (–), based on BayesR joint effects. Pink colour: the majority of variants with MAs tend to increase the trait. Dark grey: the majority of variants with MAs tend to decrease the trait. Stars: p-value for the significance of the difference in the distribution of BayesR effects between ancestral and mutant alleles at variants which were LD-clumped ($r^2 < 0.3$), '.': $p < 0.1$; '*': $p < 0.05$, '**': $p < 0.01$, '***' $p < 0.001$.

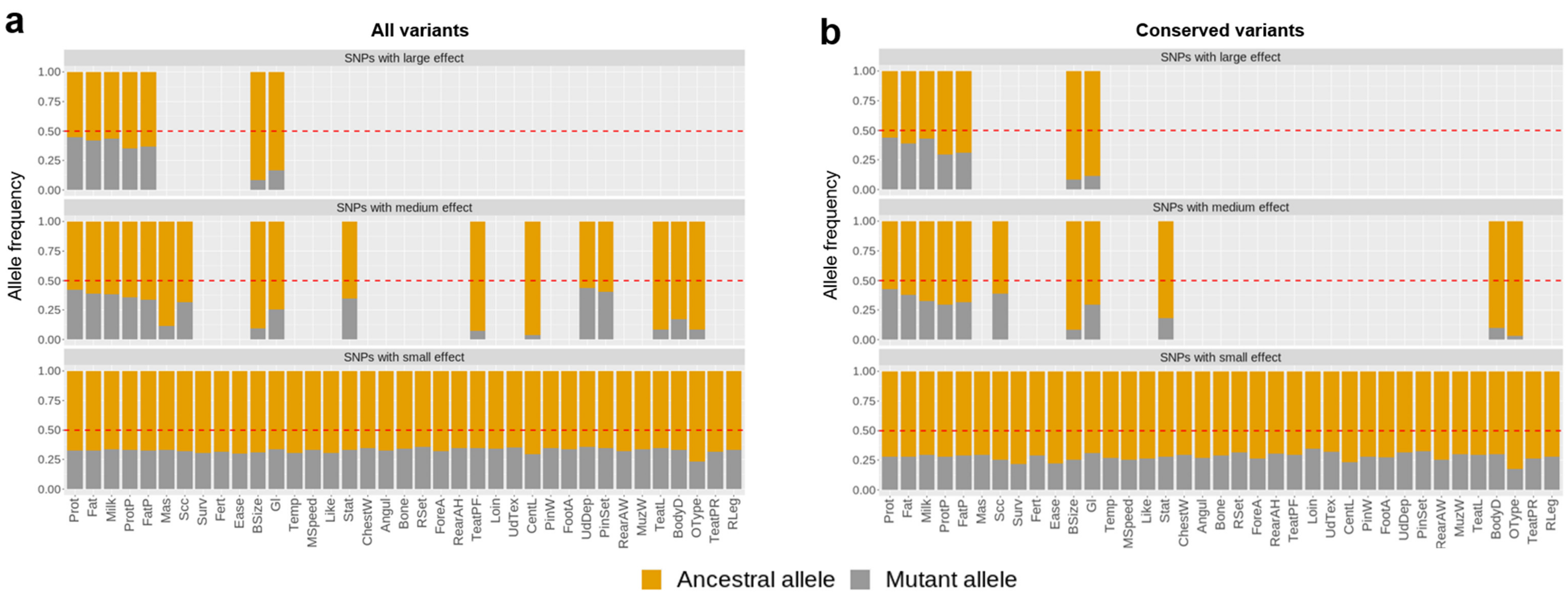

Supplementary Figure 5. Comparison of the allele frequency between ancestral and mutant alleles for variants associated with different traits. **a**: allele frequency for all analysed variants. **b**: allele frequency for variants at sites conserved across vertebrates.

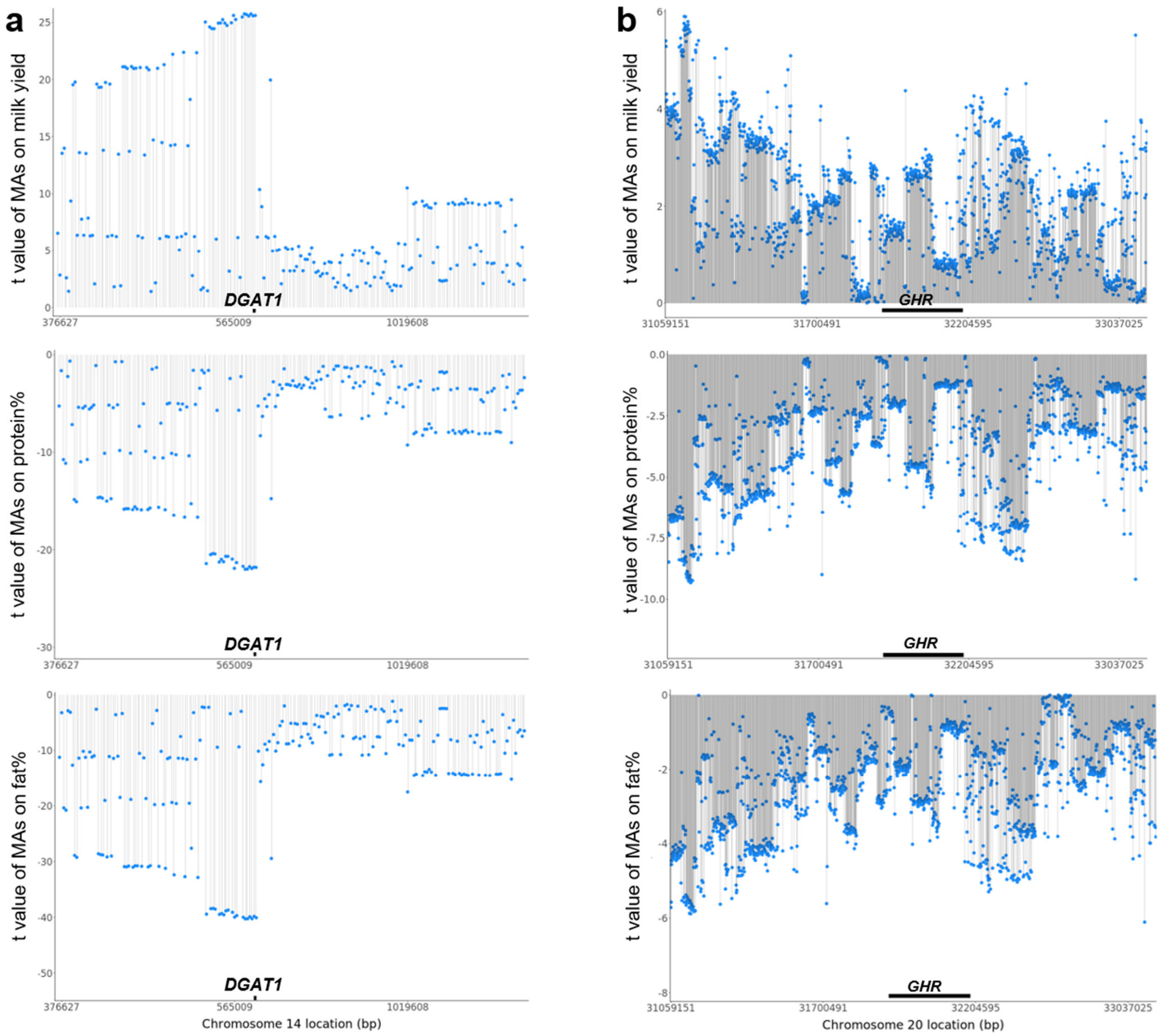

Supplementary Figure 6. GWAS t value (se/beta) for those mutant alleles (MAs) which increase milk yield but decrease protein% and fat% at *DGAT1* (a) and *GHR* (b) loci.

Supplementary Table 1. Summary of cattle traits used in the study. The 5th and 6th columns give the interpretation of each trait for its value. The term original meant the original direction of the trait when initially obtained from DataGene. The term corrected meant the direction of the trait which has been changed to improve the readability of the results.

trait full name	short name	bull.N	cow.N	larger trait value meaning (original)	larger trait value meaning (corrected)
protein yield	Prot	8097	76659	higher protein yield (good performance)	higher protein yield (good performance)
fat yield	Fat	8097	76659	higher fat yield (good performance)	higher fat yield (good performance)
milk yield	Milk	8097	76659	higher milk yield (good performance)	higher milk yield (good performance)
protein percentage	ProtP	8097	76659	higher protein percentage (good performance)	higher protein percentage (good performance)
fat percentage	FatP	8097	76659	higher fat percentage (good performance)	higher fat percentage (good performance)
mastitis	Mas	8103	77642	stronger mastitis symptom (poor performance)	stronger mastitis symptom (poor performance)
somatic cell count	Scc	8083	75429	higher somatic cell count (poor performance)	higher somatic cell count (poor performance)
survival	Surv	7147	61056	higher survival rate (good performance)	higher survival rate (good performance)
fertility	Fert	7254	56840	lower fertility (poor performance)	higher fertility (good performance)
ease (of birth)	Ease	7835	43970	more difficult to calf (poor performance)	more easier to calf (good performance)
birth size	BSize	7827	43755	larger birth size (potentially poor performance)	larger birth size (potentially poor performance)
gestation length	Gl	7181	37214	longer gestation length (potentially poor performance)	longer gestation length (potentially poor performance)
temperament	Temp	6966	37355	more aggressive (poor performance)	more docile (good performance)
milking speed	MSpeed	6966	37303	slower milking speed (poor performance)	faster milking speed (good performance)
likeability	Like	6966	37323	less favored dairy cattle (poor performance)	more favored dairy cattle (good performance)
stature	Stat	7022	45056	taller (good performance)	taller (good performance)
chest width	ChestW	7022	45056	wider chest (good performance)	wider chest (good performance)
angularity	Angul	7022	45056	angular (good performance)	angular (good performance)
bone quality	Bone	7022	45056	higher bone quality (good performance)	higher bone quality (good performance)
rear legs set	RSet	7022	45056	higher assessment score (good performance)	higher assessment score (good performance)
fore attachment	ForeA	7022	45056	higher assessment score (good performance)	higher assessment score (good performance)
rear attachment height	RearAH	7022	45056	higher assessment score (good performance)	higher assessment score (good performance)
front teat placement	TeatPF	7022	45056	higher assessment score (good performance)	higher assessment score (good performance)
loin strength	Loin	7022	45056	higher assessment score (good performance)	higher assessment score (good performance)
udder texture	UdTex	7022	45056	higher assessment score (good performance)	higher assessment score (good performance)
central ligament	CentL	7022	45055	higher assessment score (good performance)	higher assessment score (good performance)

pin width	PinW	7022	45055	higher assessment score (good performance)	higher assessment score (good performance)
foot angle	FootA	7022	45055	higher assessment score (good performance)	higher assessment score (good performance)
udder depth	UdDep	7022	45055	higher assessment score (good performance)	higher assessment score (good performance)
pin set	PinSet	7022	45054	higher assessment score (good performance)	higher assessment score (good performance)
rear attachment width	RearAW	7022	45054	higher assessment score (good performance)	higher assessment score (good performance)
muzzle width	MuzW	7022	45054	wider muzzle (good performance)	wider muzzle (good performance)
teat length	TeatL	7022	45053	longer teat (good performance)	longer teat (good performance)
body depth	BodyD	7022	45052	deeper body (good performance)	deeper body (good performance)
overall type	OType	7022	44930	higher overall score as a dairy cow (good performance)	higher overall score as a dairy cow (good performance)
rear teat placement	TeatPR	7022	44706	higher assessment score (good performance)	higher assessment score (good performance)
rear leg view	RLeg	7022	44685	higher assessment score (good performance)	higher assessment score (good performance)

Supplementary Table 2. Summary of cattle individuals used as focal species to compare with yak, sheep and camel to infer ancestral alleles.

Breed	Species	sequence coverage	BiosampleID	BioprojectID
AngusLowline	Taurus	14.0	SAMN05803884	PRJNA238491
Angus	Taurus	36.9	SAMN08473804	PRJNA432857
Abondance	Taurus	12.1	NA	NA
Alentejana	Taurus	25.1	NA	NA
BlondedAquitaine	Taurus	26.6	NA	NA
Crossbreed	Other	15.1	SAMN02843106	PRJNA176557
Beefmaster	Taurus_Indicus	26.9	SAMN05216053	PRJNA324822
Boran	Indicus	13.8	NA	NA
Brahman	Indicus	49.5	NA	NA
Shorthorn	Taurus	21.8	SAMN05788509	PRJNA343262
BrownSwiss	Taurus	34.4	NA	NA
OrigBraunvieh	Taurus	37.0	NA	NA
Charolais	Taurus	21.4	NA	NA
Devon	Taurus	10.4	SAMN02841105	PRJNA176557
Friesian	Taurus	14.3	NA	NA
Droughtmaster	Taurus_Indicus	19.6	NA	NA
Dexter	Taurus	13.6	SAMN05803883	PRJNA238491
BeltedGalloway	Taurus	13.3	SAMN05803879	PRJNA238491
Guernsey	Taurus	13.5	NA	NA
Gelbvieh	Taurus	21.1	NA	NA
Hariana	Indicus	27.7	NA	NA
HerefordMiniature	Taurus	11.6	SAMN05803882	PRJNA238491
Hereford	Taurus	45.5	NA	NA
Hinterwaelder	Taurus	10.4	NA	NA
Highland	Taurus	31.5	NA	NA
Holstein	Taurus	59.1	SAMN08612438	PRJNA431934
HolsteinFriesian	Taurus	28.3	NA	NA
IranAdmixed	Taurus_Indicus	12.2	SAMEA2358281	PRJEB1829
Jersey	Taurus	31.6	SAMN08612544	PRJNA431934
JerseyLimousin	Taurus	31.5	NA	NA
Kholmogory	Taurus	15.1	NA	NA
Lagune	Other	20.0	NA	NA
Limousin	Taurus	29.9	NA	NA
MurrayGrey	Taurus	14.3	NA	NA
Montbeliarde	Taurus	27.7	SAMEA3390170	PRJEB9343
NDama	Taurus	16.4	SAMN05788519	PRJNA343262
Normande	Taurus	30.8	SAMEA3390191	PRJEB9343
Piemontese	Taurus	27.7	NA	NA
Pinzgauer	Taurus	32.8	NA	NA
AngusRed	Taurus	22.1	NA	NA
Illawarra	Taurus	16.8	NA	NA
GermanRedAngler	Taurus	11.7	NA	NA
TraditionalDanishRed	Taurus	11.6	NA	NA

ModernDanishRed	Taurus	10.7	NA	NA
FinnishAyrshire	Taurus	19.3	NA	NA
NorwegianRed	Taurus	33.4	SAMN09510423	PRJNA477833
SwedishRed	Taurus	11.3	NA	NA
RougeDesPres	Taurus	12.8	NA	NA
MaineAnjou	Taurus	22.7	SAMN05788562	PRJNA343262
HolsteinRed	Taurus	12.2	NA	NA
Romangola	Taurus	11.3	NA	NA
SantaGertrudis	Taurus_Indicus	19.1	NA	NA
JapaneseNative	Other	11.7	SAMD00013963	PRJDA48395
Hanwoo	Taurus	11.3	SAMN02225732	PRJNA210523
Korean	Other	22.5	SAMN02225750	PRJNA210519
HolsteinHereford	Taurus	16.6	SAMN10598571	PRJNA494431
SouthAnatolianRed	Taurus	14.1	NA	NA
Shaiwal	Other	17.0	NA	NA
Sikias	Other	16.7	NA	NA
Simmental	Taurus	33.9	NA	NA
SimmentalFleckviehPezzatarossa	Taurus	14.2	NA	NA
SwissFleckvieh	Taurus	17.2	NA	NA
RedSindhi	Indicus	12.9	NA	NA
Somba	Other	26.3	NA	NA
Stabilizer	Taurus	11.8	NA	NA
Tarentaise	Taurus	12.7	NA	NA
TyroleanGrey	Taurus	25.3	NA	NA
Tharparkar	Indicus	13.5	NA	NA
Tuli	Taurus	11.2	NA	NA
Tuxer	Taurus	43.8	NA	NA
UgandaAdmixed	Other	11.2	SAMEA2065738	PRJEB1829
Mongolian	Other	11.3	SAMN06698981	PRJNA379859
Kazakh	Taurus	11.9	SAMN06698986	PRJNA379859
JiaxianRed	Other	12.6	SAMN06699017	PRJNA379859
Brangus	Indicus	21.0	SAMN05216058	PRJNA324822
Salers	Taurus	14.0	SAMN05216064	PRJNA324822
TexasLonghorn	Taurus	10.7	SAMN05216082	PRJNA324822
Chianina	Taurus	13.5	SAMN05216092	PRJNA324822
Braunvieh	Taurus	11.5	SAMN05216096	PRJNA324822
Corriente	Taurus	12.4	SAMN05216099	PRJNA324822
ChaidamuYellow	Taurus_Indicus	10.9	SAMN06698977	PRJNA379859
TibetanYellow	Other	11.9	SAMN06699003	PRJNA379859
Xuanhan	Other	10.4	SAMN06699039	PRJNA379859
Wannan	Other	10.9	SAMN06699048	PRJNA379859
Dabieshan	Taurus	10.5	SAMN06699055	PRJNA379859
Wenshan	Other	11.3	SAMN07622461	PRJNA379859
Tibetan	Other	13.3	SAMN07622466	PRJNA379859
Gir	Indicus	59.2	SAMN08225763	PRJNA427256
FriesianJersey	Taurus	21.8	SAMN08810150	PRJNA446068

BelgianBlue	Taurus	13.9	SAMN09379816	PRJNA474946
Vorderwaelder	Taurus	16.1	NA	NA
Vosgienne	Taurus	12.8	NA	NA
Wagyu	Taurus	15.1	NA	NA
Composite	Taurus_Indicus	41.6	NA	NA
HolsteinCharolais	Taurus	28.8	NA	NA
Lowline	Other	25.7	NA	NA
MAARCTwinner	Taurus	17.3	NA	NA
Yakut	Taurus	12.1	NA	NA

Supplementary Table 3. Average proportion (%) of variance of cow traits explained by variants with large (L, GWAS p-value < 5e-8 in both sexes), medium (M, GWAS p-value < 5e-5 and p-value > 5e-8 in both sexes) and small (S, GWAS p-value < 5e-2 and p-value > 5e-5 in both sexes) effects, with their standard effort (se). The number variants in each group is also given.

trait order	trait name	Effect size	Variance %	se	No. of variants
tr01	Prot	S	0.010%	0.0001%	35256
tr01		M	0.063%	0.0015%	598
tr01		L	0.346%	0.0169%	502
tr02	Fat	S	0.012%	0.0001%	29591
tr02		M	0.073%	0.0014%	1171
tr02		L	0.358%	0.0138%	1879
tr03	Milk	S	0.015%	0.0001%	39173
tr03		M	0.070%	0.0016%	1449
tr03		L	0.417%	0.0200%	1658
tr04	ProtP	S	0.015%	0.0001%	52472
tr04		M	0.059%	0.0006%	4554
tr04		L	0.254%	0.0086%	2616
tr05	FatP	S	0.014%	0.0001%	30923
tr05		L	0.424%	0.0187%	2944
tr05		M	0.064%	0.0011%	1948
tr06	Mas	S	0.009%	0.00002%	31021
tr06		M	0.030%	0.0002%	3
tr07	Scc	S	0.009%	0.00002%	41740
tr07		M	0.029%	0.0017%	36
tr08	Surv	S	0.010%	0.00004%	10878
tr09	Fert	S	0.012%	0.0001%	17971
tr10	Ease	S	0.014%	0.00004%	10373
tr11	BSize	S	0.015%	0.0001%	11208
tr11		M	0.060%	0.0043%	6
tr11		L	0.109%	0.0040%	2
tr12	Gl	S	0.023%	0.0001%	26914
tr12		M	0.164%	0.0038%	505
tr12		L	0.228%	0.0359%	26
tr13	Temp	S	0.016%	0.00004%	14243
tr14	MSpeed	S	0.018%	0.0001%	19438
tr15	Like	S	0.017%	0.00005%	14215
tr16	Stat	S	0.018%	0.0001%	27049
tr16		M	0.140%	0.0039%	82
tr17	ChestW	S	0.014%	0.00005%	16667
tr18	Angul	S	0.014%	0.00004%	16349
tr19	Bone	S	0.015%	0.0001%	19792
tr20	RSet	S	0.014%	0.00004%	15116
tr21	ForeA	S	0.014%	0.00004%	16152
tr22	RearAH	S	0.014%	0.00005%	19465
tr23	TeatPF	S	0.016%	0.0001%	25779
tr23		M	0.040%	0.0000%	2
tr24	Loin	S	0.014%	0.00004%	15453

tr25	UdTex	S	0.014%	0.00004%	13962
tr26	CentL	S	0.014%	0.00004%	18823
tr26		M	0.065%	NA	1
tr27	PinW	S	0.014%	0.00004%	20783
tr28	FootA	S	0.014%	0.00004%	16767
tr29	UdDep	S	0.016%	0.00005%	27364
tr29		M	0.070%	0.0025%	7
tr30	PinSet	S	0.016%	0.00005%	25875
tr30		M	0.042%	0.0014%	3
tr31	RearAW	S	0.015%	0.00005%	18050
tr32	MuzW	S	0.015%	0.00004%	19731
tr33	TeatL	S	0.018%	0.0001%	34086
tr33		M	0.087%	0.0436%	2
tr34	BodyD	S	0.016%	0.0001%	22536
tr34		M	0.099%	0.0137%	14
tr35	OType	S	0.015%	0.00004%	24340
tr35		M	0.042%	0.0003%	15
tr36	TeatPR	S	0.015%	0.00004%	21500
tr37	RLeg	S	0.014%	0.00004%	11063

Supplementary Table 4. Summary of the direction of effects of MAs with large effects on milk production on traits on the expression of genes in milk cells [1,2].

Trait	N.mut_neg_traitSNP	N.best_cisSNP	N.mut_neg_cisSNP	prop.mut_neg_cisSNP
Prot	281	71	49	69.0%
Fat	929	147	81	55.1%
Milk	902	132	39	29.5%
Prot%	1561	235	141	60.0%
Fat%	1587	159	108	67.9%
trait	N.mut_pos_traitSNP	N.best_cisSNP	N.mut_pos_cisSNP	prop.mut_pos_cisSNP
Prot	221	111	78	70.3%
Fat	950	92	51	55.4%
Milk	756	115	55	47.8%
Prot%	1054	201	103	51.2%
Fat%	1357	141	90	63.8%

N.mut_neg_traitSNP: number of variants where the MA decreases traits at GWAS p < 5e-8.
N.best_cisSNP: number of the best cis eQTL variants (smallest p-value in eQTL mapping, ±1Mb of the gene) in milk cells.
N.mut_neg_cisSNP: number of the best cis eQTL variants where the mutant allele decreases the expression of genes.
prop.mut_neg_cisSNP: proportion of the best cis eQTL variants where the mutant allele decreases the expression of genes.
N.mut_pos_traitSNP: number of variants that the MA increases traits at GWAS p < 5e-8.
N.mut_pos_cisSNP: number of the best cis eQTL variants where the mutant allele increases the expression of genes.
prop.mut_pos_cisSNP: proportion of the best cis eQTL variants where the mutant allele increases the expression of genes.

Supplementary References

1 Xiang, R. *et al.* Genome variants associated with RNA splicing variations in bovine are extensively shared between tissues. *BMC Genomics* **19**, 521, doi:10.1186/s12864-018-4902-8 (2018).

2 Chamberlain, A. *et al.* in *11th world congress on genetics applied to livestock production (WCGALP). Auckland, New Zealand: Volume Molecular Genetics.* 254.
