# Supplementary material for: Mutant alleles differentially shape fitness and other complex traits in cattle
Source: Commun Biol. 2021 Dec 2;4:1353. doi: 10.1038/s42003-021-02874-9 (PMC8640064; doi:10.1038/s42003-021-02874-9)
Supplement: Supplementary file 3 — Description of Additional Supplementary Files [file 42003_2021_2874_MOESM3_ESM.pdf]

Description of Additional Supplementary Files

File name: Supplementary Data 1
Description: Pleiotropic effects of selected mutant alleles (MAs).

File name: Supplementary Data 2
Description: Information on 1720 cattle from the 1000 Bull Genome Project used for validation analysis.
