# Supplementary material for: Mutant alleles differentially shape fitness and other complex traits in cattle
Source: Commun Biol. 2021 Dec 2;4:1353. doi: 10.1038/s42003-021-02874-9 (PMC8640064; doi:10.1038/s42003-021-02874-9)
Supplement: Supplementary file 6 — Reporting Summary [file 42003_2021_2874_MOESM6_ESM.pdf]

nature research

Corresponding author(s): Ruidong Xiang

Last updated by author(s): Oct 27, 2021

Reporting Summary

Nature Research wishes to improve the reproducibility of the work that we publish. This form provides structure for consistency and transparency in reporting. For further information on Nature Research policies, see our Editorial Policies and the Editorial Policy Checklist.

Statistics

For all statistical analyses, confirm that the following items are present in the figure legend, table legend, main text, or Methods section.

n/a	Confirmed	
☐	☒	The exact sample size (n) for each experimental group/condition, given as a discrete number and unit of measurement
☐	☒	A statement on whether measurements were taken from distinct samples or whether the same sample was measured repeatedly
☐	☒	The statistical test(s) used AND whether they are one- or two-sided<br>*Only common tests should be described solely by name; describe more complex techniques in the Methods section.*
☐	☒	A description of all covariates tested
☐	☒	A description of any assumptions or corrections, such as tests of normality and adjustment for multiple comparisons
☐	☒	A full description of the statistical parameters including central tendency (e.g. means) or other basic estimates (e.g. regression coefficient) AND variation (e.g. standard deviation) or associated estimates of uncertainty (e.g. confidence intervals)
☐	☒	For null hypothesis testing, the test statistic (e.g. F, t, r) with confidence intervals, effect sizes, degrees of freedom and P value noted<br>*Give P values as exact values whenever suitable.*
☐	☒	For Bayesian analysis, information on the choice of priors and Markov chain Monte Carlo settings
☐	☒	For hierarchical and complex designs, identification of the appropriate level for tests and full reporting of outcomes
☐	☒	Estimates of effect sizes (e.g. Cohen's d, Pearson's r), indicating how they were calculated

Our web collection on statistics for biologists contains articles on many of the points above.

Software and code

Policy information about availability of computer code

Data collection	NA.
Data analysis	The probability of ancestral allele assignment used the software published by ref [19]. The linear mixed model used GCTA ref [32]and MTG2 ref [33]. The Bayesian analysis used BayesR ref [39]. The R code of estimating heterozygosity across conserved sites will be made public upon publication.

For manuscripts utilizing custom algorithms or software that are central to the research but not yet described in published literature, software must be made available to editors and reviewers. We strongly encourage code deposition in a community repository (e.g. GitHub). See the Nature Research guidelines for submitting code & software for further information.

Data

Policy information about availability of data

All manuscripts must include a data availability statement. This statement should provide the following information, where applicable:

- Accession codes, unique identifiers, or web links for publicly available datasets
- A list of figures that have associated raw data
- A description of any restrictions on data availability

Our predictions of cattle ancestral alleles for those 42,573,455 sites have been made publicly available at: https://figshare.com/s/dd5985b76a413b56106b. Multiple alignment data used to determine cattle ancestral alleles are publicly available via Ensembl EPO pipeline (http://asia.ensembl.org/info/genome/compara/multiple_genome_alignments.html). Australian farmers and DataGene Australia (http://www.datagene.com.au/) are owners and custodians of the raw phenotype and genotype data of Australian dairy animals. Access to these data for research requires permission from DataGene under a Data Use Agreement. The DNA sequence data as part of the 1000 Bull Genomes Consortium 21-23 are available to consortium members and the membership is open. Sequence data of 1832 samples from the 1000 Bull Genome Project have been made publicly available at: https://www.ebi.ac.uk/eva/?eva-study=PRJEB42783. The gene expression data is

publicly available (NCBI SRA SRP111067). In addition: 1. The summary data of the effect direction and effect category of those 7.9M sequence variants for which the ancestral alleles can be assigned is published at https://figshare.com/s/ef020d948523c31c0e67; 2. The allele frequency of mutant alleles of those 7.9M sequence variants for which the ancestral alleles can be assigned for the Holstein and Jersey cattle from the 1000 Bull Genome Project is published at https://figshare.com/s/20154b1d8e60e012e532; 3. The coordinates of conserved sites analysed in the manuscript is published at: https://figshare.com/s/df9d3662f8f7fb8e72da. Other supporting data are shown in the supplementary materials of the current manuscript. Source data of figures can be found at https://figshare.com/s/25a41d9cc33d72a11fe4.

Field-specific reporting

Please select the one below that is the best fit for your research. If you are not sure, read the appropriate sections before making your selection.

☒ Life sciences ☐ Behavioural & social sciences ☐ Ecological, evolutionary & environmental sciences

For a reference copy of the document with all sections, see nature.com/documents/nr-reporting-summary-flat.pdf

Life sciences study design

All studies must disclose on these points even when the disclosure is negative.

Sample size	We used all data available to us from DataGene Australia (https://datagene.com.au/).
Data exclusions	NA.
Replication	We included external data from the 1000 Bull Genomes consortium to replicate our results.
Randomization	NA.
Blinding	Investigators were blinded when collecting data.

Reporting for specific materials, systems and methods

We require information from authors about some types of materials, experimental systems and methods used in many studies. Here, indicate whether each material, system or method listed is relevant to your study. If you are not sure if a list item applies to your research, read the appropriate section before selecting a response.

Materials & experimental systems

n/a	Involved in the study	
☒	☐	Antibodies
☒	☐	Eukaryotic cell lines
☒	☐	Palaeontology and archaeology
☒	☐	Animals and other organisms
☒	☐	Human research participants
☒	☐	Clinical data
☒	☐	Dual use research of concern

Methods

n/a	Involved in the study	
☒	☐	ChIP-seq
☒	☐	Flow cytometry
☒	☐	MRI-based neuroimaging
